# The Brain-Muscle Axis in Neurodegeneration: Linking Dementia, Sarcopenia, and Exercise-Mediated Neuroprotection

**DOI:** 10.3390/ijms27167249

**Published:** 2026-08-14

**Authors:** Garima Paul, Mehek Sharma, Diana M. Asante, Deborah A. Jehu, Mohammad Badruzzaman Khan, Carlos Isales, Sadanand Fulzele

**Affiliations:** 1 Department of Medicine, Augusta University, Augusta, GA 30912, USA; gpaul@augusta.edu (G.P.); mesharma@augusta.edu (M.S.); cisales@augusta.edu (C.I.); 2 Department of Cell Biology and Anatomy, Augusta University, Augusta, GA 30912, USA; dasante@augusta.edu; 3 Department of Community & Behavioral Health Sciences, Institute of Public and Preventive, Augusta, GA 30912, USA; djehu@augusta.edu; 4 Department of Neurology, Augusta University, Augusta, GA 30912, USA; mkhan@augusta.edu; 5 Center for Healthy Aging, Augusta University, Augusta, GA 30912, USA; 6 Department of Neuroscience & Regenerative Medicine, Augusta University, Augusta, GA 30912, USA

**Keywords:** sarcopenia, dementia, exercise, muscle

## Abstract

Age-related dementia is commonly associated with several secondary complications such as sarcopenia. To date, the correlation between dementia and sarcopenia is not completely understood. This review summarizes the correlation between dementia and sarcopenia using human clinical and observational studies. We also summarize the impact of exercise on these two conditions. We performed a narrative search with systematic elements using PubMed, Embase, Web of Science, and Google Scholar. In this review, we included studies that performed dementia assessments using appropriate methods (e.g., Mini-Mental State Examination, neuropsychological testing, brain MRI, or clinical dementia rating scales) and validated sarcopenia criteria (e.g., European Working Group on Sarcopenia in Older People). We included a total of nine studies consisting of 5217 participants (mean age 63.1–87.6 years). Of these, participants had dementia-only or mixed cognitive cohorts. Depending on diagnostic criteria, the prevalence of sarcopenia among individuals with dementia ranged from 6.8% to 68.8%. Most of the studies showed a positive association between dementia and sarcopenia. Interestingly, the higher the cognitive impairment, the higher the physical decline and signs of sarcopenia. As expected, any kind of physical activity (e.g., aerobic exercise, walking, and Tai Chi) improves cognitive function and brain health. Overall, the current literature supports that dementia is associated with increased risk of sarcopenia, supporting a brain-muscle axis in neurodegeneration. Physical activity appears to be a key modifiable factor with benefits for both cognitive and musculoskeletal health. Further longitudinal and mechanistic studies are needed to clarify causality and underlying pathways.

## 1. Introduction

Improved health care in the United States from 2000 to 2019 increased life expectancy by 2.3 years [1]. This has led to an increasing prioritization of the prevention and management of chronic diseases, particularly neurodegenerative disorders such as dementia. The prevalence of dementia increased sharply with age, approximately by 5% in individuals aged 71–79 years to 24.2% in those aged 80–89 years [2]. Increased prevalence of dementia puts a significant emotional and financial burden on patients, caregivers, and healthcare systems. The average annual cost of caregiving per dementia patient in 2019 was approximately $42,000 [3], which is projected to increase in the coming years.

The increase in the dementia patient population is due to advanced imaging diagnostic capabilities, including neuroimaging techniques such as magnetic resonance imaging (MRI) and positron emission tomography (PET). Furthermore, emerging blood- and cerebrospinal fluid biomarkers such as phosphorylated tau (p-tau181 and p-tau217), the amyloid-β42/40 ratio, neurofilament light chain (NfL), and glial fibrillary acidic protein (GFAP) improve the early detection of Alzheimer’s disease and related dementias [4]. Due to this, chronic diseases have decreased the mortality rates associated with dementia, leading to the emergence of secondary complications and comorbidities. These comorbidities potentially accelerate the progression of dementia, exacerbating the underlying condition [5,6,7,8]. Alzheimer’s disease is typically associated with progressive decline beginning with short-term memory impairment and advancing to severe cognitive dysfunction, personality changes, and psychosis [6].

Along with the cognitive impairment, patients also exhibit other symptoms such as gait disturbances, including ataxia, as well as features of parkinsonism and sleep-related disorders [9,10]. The pathogenesis of dementia involves several mechanisms, including vascular and nonvascular causes. Vascular causes account for 10–20% of dementia [11] and often involve cerebral microvascular dysfunction. Blood–brain barrier (BBB) disruption is a well-known pathological feature that follows ischemic injury, allowing toxic molecules such as fibrinogen, plasminogen, and thrombin to cross and accumulate in the brain, even in the absence of beta-amyloid [12].

Dementia patients are also associated with multiple comorbidities, including diabetes, peripheral vascular disease, cerebrovascular disease, hypertension, and reduced musculoskeletal function characterized by reduced muscle mass and strength [13]. Recent preclinical studies support the concept that neurodegeneration extends beyond the central nervous system, disrupting systemic homeostasis and contributing to peripheral tissue pathology, including skeletal muscle decline [14]. This brain–muscle interaction may exacerbate functional decline and accelerate cognitive dysfunction, supporting the concept of dementia as a multisystem disorder [15]. Most importantly, individuals with dementia exhibit particularly sedentary behavior due to progressive cognitive decline, impaired mobility, and reduced independence in activities of daily living, creating a vicious cycle that accelerates sarcopenia progression, frailty, and potentially worsens neurocognitive outcomes [16]. Given this evolving paradigm, there is a need to better characterize the clinical and mechanistic links between dementia and sarcopenia. This narrative review summarizes recent clinical evidence demonstrating the bidirectional relationship between dementia and decreased muscle mass and function. Our study also explores the role of exercise in modulating cognitive and muscular outcomes in this population.

## 2. Methods

### 2.1. Literature Search Strategy

This narrative review incorporated systematic elements to provide a structured and comprehensive synthesis of the literature on the relationship between dementia, sarcopenia, and exercise-mediated neuroprotection. This review was performed in accordance with the Preferred Reporting Items for Systematic Reviews and Meta-Analyses (PRISMA) guidelines and was registered in the International Prospective Register of Systematic Reviews (PROSPERO) database (Registration number: CRD420251005418). A structured search strategy with systematic elements was used to evaluate the relationship between dementia, sarcopenia, and exercise-mediated neuroprotection. Electronic databases, including PubMed, Google Scholar, Embase, and Web of Science, were searched from January 2025 to July 2025. The search strategy incorporated combinations of Medical Subject Headings (MeSH) terms and keywords related to dementia, sarcopenia, and exercise. The core search structure was as follows: The search string was as follows: (“Amyloid B-plaque clearance” OR “muscle loss” OR “cognitive impairment” OR “muscle atrophy” OR “BDNF” AND “Dementia” OR “Exercise” AND “Alzheimer’s Disease” OR “Humans” OR (“Alzheimer’s Disease” OR “Dementia”) AND (“Sarcopenia”). Studies identified through this systematic search formed the basis of the clinical evidence synthesis presented in the dementia-sarcopenia section.

In addition, a narrative literature synthesis was performed to contextualize the clinical findings and explore potential mechanisms underlying the brain-muscle axis and the role of exercise-mediated neuroprotection. In addition, mechanistic studies (such as preclinical animal models and studies examining exercise-related effects on cognitive function, neurotrophic signaling, neuroinflammation, and biomarkers of neurodegeneration) were identified through targeted literature review. They were not subject to the systematic screening and eligibility process described above. These studies were selected based on their relevance to the mechanistic framework and clinical context of the review.

### 2.2. Study Selection and Eligibility Criteria

For the systematic component, studies were screened in two stages. First, titles and abstracts were screened for relevance to the review topic. Second, full-text articles were assessed for eligibility based on predefined inclusion and exclusion criteria. Studies were included if they met the following criteria: (1) studies investigating the effect of dementia or Alzheimer’s Disease on the development of sarcopenia in human models as well as studies on how exercise influences Alzheimer’s Disease and Dementia, (2) studies reporting original data, (3) studies published in peer-reviewed journals, and (4) studies conducted in English (5) international and regional studies (6) Studies within the last 25 years. Exclusion criteria were: (1) studies not relevant to the topic of interest, (2) studies considered qualitative studies or duplicate publications, (3) published in languages other than English, and (4) studies with confounding factors. All relevant articles published up to July 2025 were considered for inclusion.

The exercise and mechanistic literature was not subject to the same systematic eligibility criteria. Instead, relevant human exercise studies and preclinical mechanistic studies were selected narratively to examine potential pathways linking skeletal muscle activity with brain health and to contextualize the clinical association between dementia and sarcopenia.

### 2.3. Data Extraction

We extracted data based on study design, sample size, participant demographics, dementia or cognitive assessment, sarcopenia diagnostic criteria, outcome measures, and key findings. The sarcopenia definitions were based on European Working Group on Sarcopenia in Older People (EWGSOP1 and EWGSOP2) criteria wherever applicable. These studies were synthesized qualitatively to characterize the direction and strength of the clinical association between dementia and sarcopenia. Studies included in the narrative review of mechanistic and exercise studies were carefully reviewed for their relevance to neurotrophic signaling, neuroinflammation, mitochondrial function, cognitive outcomes, and musculoskeletal health.

## 3. Results and Discussion

**Figure 1:**
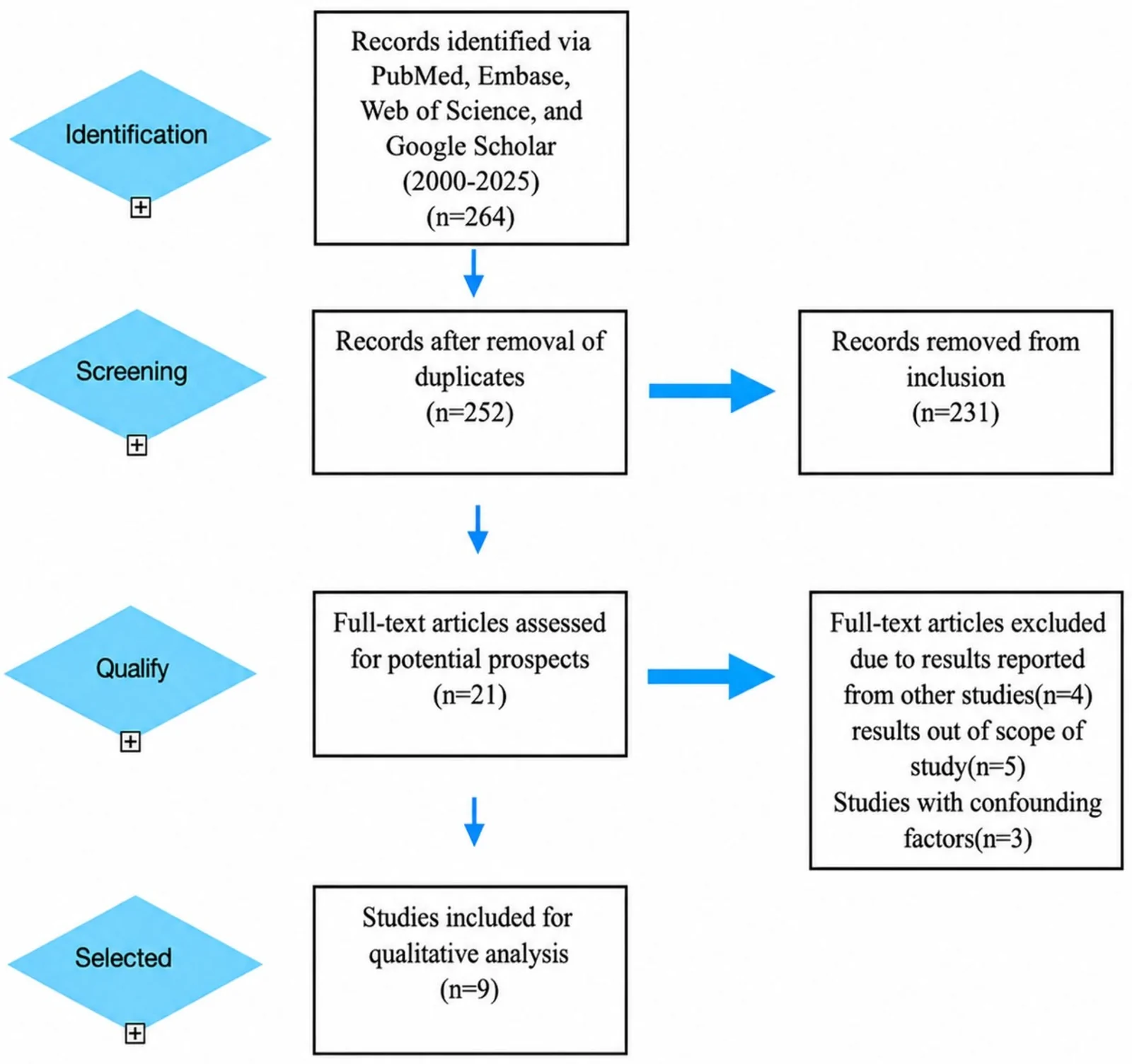
PRISMA flow diagram of the systematic identification and selection of clinical studies evaluating the association between dementia and sarcopenia. The 9 studies included in the systematic clinical evidence synthesis were distinct from the studies discussed in the subsequent narrative synthesis of exercise and mechanistic evidence.

Our comprehensive literature search identified 264 records, of which 252 remained after removal of duplicates. Following initial screening, 21 full-text articles were assessed for eligibility. Of these, 4 were excluded due to lack of original data, 5 were removed for being outside the study scope, and 3 were excluded due to confounding factors or inappropriate measurement of dementia or sarcopenia. Ultimately, 9 studies were included in the final analysis (Figure 1). All studies were published between 2000 and 2025, ensuring the inclusion of recent research in the field. A total of 5217 participants were analyzed, with mean ages ranging from 63.1 to 87.6 years, including both dementia-only cohorts and mixed populations with defined cognitive subgroups. We included multicenter, cross-sectional, observational, longitudinal, and population-based studies (Table 1). We observed that the prevalence of sarcopenia varied considerably across studies (6.8–68.8%). This variability is mainly due to the differences in study populations, dementia severity, and diagnostic criteria used to define both dementia and sarcopenia.

Despite this variability, the overall pattern across the included literature suggested that individuals with dementia generally had a higher prevalence of sarcopenia, reduced muscle strength, and poorer physical performance than cognitively normal controls. Importantly, because most included studies were cross-sectional or observational, these findings demonstrate an association rather than a causal relationship. In addition, studies evaluating physical activity interventions, including aerobic exercise, walking, and Tai Chi, reported improvements in cognitive function and biomarkers of brain health, supporting exercise as a potential strategy to preserve cognitive function while simultaneously promoting musculoskeletal health.

### 3.1. Sarcopenia as a Comorbidity of Clinical Dementia

Age is a significant risk factor for sarcopenia, defined as the progressive loss of skeletal muscle mass and function [17]. With age, individuals become more sedentary, which contributes to the deterioration of muscle strength and endurance, exacerbating the onset and progression of sarcopenia [18]. Muscle biopsies from older individuals demonstrate a 25–35% reduction in muscle mass, with a higher proportion of fat and connective tissue [19]. The loss of type 2 muscle fibers (fast-twitch) in both quantity and magnitude is particularly prevalent in age-related sarcopenia [20]. While these age-related changes are well established, emerging evidence suggests that neurodegenerative processes may further accelerate muscle decline beyond what is expected with aging alone. In support of this, several longitudinal, case–control, and observational studies in humans have shown an association between dementia and the development or progression of sarcopenia [21] (Table 1).

The evidence is derived predominantly from cross-sectional and observational studies, which generally report a higher burden of sarcopenia or impaired muscle function among individuals with cognitive impairment. However, the magnitude of this association varies across populations and according to how sarcopenia and cognitive impairment are defined. In a cross-sectional cohort of patients with Alzheimer’s disease, Bramato et al. (2022) reported a sarcopenia prevalence of 23.8% [22]. In contrast, Özsürekci et al. (2019) [23] evaluated 148 individuals with Alzheimer’s disease across different disease stages. They found that sarcopenia was associated with dysphagia, but sarcopenia prevalence did not differ substantially across stages of Alzheimer’s disease [23]. This contrasts with studies suggesting a progressive increase in sarcopenia with worsening cognitive impairment and indicates that the relationship between dementia severity and sarcopenia may depend on the clinical outcome examined. The association between sarcopenia and dysphagia also highlights the potential contribution of impaired nutrition and swallowing function to muscle loss, which may represent an important mediator or confounder rather than a direct consequence of neurodegeneration. Ogawa et al. (2018) similarly examined sarcopenia across stages of Alzheimer’s disease, categorizing participants as cognitively normal, early AD, or moderate AD [24]. Sarcopenia was assessed using measures of upper- and lower-extremity muscle strength and mass, as well as gait speed. Prevalence increased from 13% in cognitively normal participants to 41% in early AD and 47% in moderate AD, with AD groups demonstrating significantly higher prevalence than controls (*p* < 0.05 or *p* < 0.01). Although these findings suggest greater sarcopenic burden among individuals with AD, the plateau in prevalence between mild cognitive impairment (MCI) and moderate disease suggests that the relationship may not be strictly linear across disease stages.

Similarly, Sugimoto et al. evaluated 418 participants with normal cognition, MCI, or Alzheimer’s disease, comprising 35 individuals with normal cognition, 50 with MCI, and 343 with AD [25]. Sarcopenia was assessed using handgrip strength, skeletal muscle mass, and serum 25-hydroxyvitamin D, while cognitive status was evaluated using the Montreal Cognitive Assessment. Sarcopenia prevalence increased across cognitive groups, from 8.6% in cognitively normal participants to 12.5% in those with MCI and 23.3% in those with AD. This graded association is consistent with greater sarcopenic burden accompanying greater cognitive impairment; however, because these comparisons were cross-sectional, they cannot establish whether cognitive decline preceded muscle loss or whether progressive muscle dysfunction contributed to cognitive deterioration. Factors associated with sarcopenia included lower BMI, older age, low vitamin D levels among women, and lower vitality [25]. These findings also highlight the potential contribution of nutritional, demographic, and functional factors to the observed association rather than indicating a direct effect of neurodegeneration alone.

Longitudinal and population-based evidence provides greater insight into the temporal relationship between neurodegenerative pathology and sarcopenia, although such evidence remains limited. Ceolin et al. (2025) conducted a 12-year population-based study of 2291 adults aged 60 years or older, examining associations between Alzheimer’s disease-related blood biomarkers and sarcopenia development and progression [26]. Muscle strength was evaluated using chair-stand and handgrip tests, and muscle mass using calf circumference. Alzheimer’s disease-related blood biomarkers measured included Aβ42, total tau, p-tau181, NfL, and GFAP. Among participants, 522 (22.8%) had probable sarcopenia and 161 (7.0%) had confirmed sarcopenia. Higher p-tau181 and NfL concentrations were associated with sarcopenic outcomes, with reported odds ratios of 1.24 (95% CI, 1.09–1.42; *p* = 0.002) and 1.56 (95% CI, 1.30–1.91; *p* < 0.001), respectively. These findings provide additional evidence of a temporal association between neurodegenerative biomarkers and subsequent sarcopenic outcomes, but the observational design does not establish a direct relation between these two conditions. Residual confounding by age, comorbidity, nutritional status, physical activity, or frailty may also contribute to the observed associations. Thus, the relationship between neurodegenerative biomarkers and sarcopenia raises the possibility of shared biological pathways but does not distinguish between direct effects of neurodegeneration, downstream effects of reduced activity, and common upstream risk factors.

The clinical studies also differ substantially in how sarcopenia was defined. Several studies used EWGSOP or EWGSOP2 criteria incorporating muscle mass, grip strength, and physical performance, whereas others used AWGS criteria or examined individual components of sarcopenia separately. These methodological differences likely contributed to the wide range of reported prevalence. For example, Sarabia et al. (2014) reported a sarcopenia prevalence of 68.8% among institutionalized individuals with dementia [27], whereas Bramato et al. (2022) reported a prevalence of 23.8% in a different Alzheimer’s disease cohort [22]. These estimates should therefore not be interpreted as directly comparable measures of disease frequency. The distinction between sarcopenia and isolated impairments in muscle strength or physical performance is also important. In a cohort of 731 community-dwelling older adults, Huang et al. [28] found that global cognitive impairment was not significantly associated with sarcopenia as a composite diagnosis. However, cognitive impairment was associated with reduced muscle strength and physical performance. Sarcopenia was additionally associated with poorer verbal fluency (OR 3.96, *p* = 0.0006) [28]. These findings suggest that cognitive impairment may relate differently to individual components of muscle dysfunction than to sarcopenia defined as a composite syndrome.

Potential confounding and mediation further complicate interpretation of the observed association. Nutritional status and appetite were repeatedly identified as relevant factors. Sarabia et al. (2014) reported an association between malnutrition and sarcopenia among individuals with dementia [27], whereas Kimura et al. (2018) demonstrated that lower appetite scores were associated with sarcopenia in patients with MCI [29]. These studies suggested that reduced food intake and nutritional deficiencies might partially mediate the relationship between cognitive impairment and muscle loss rather than representing independent effects of neurodegeneration. Similarly, reduced physical activity, frailty, low BMI, depression, and multimorbidity may contribute to both cognitive and musculoskeletal decline. Endo et al., for example, found no statistically significant association between dementia and sarcopenia in their cohort, while depression was associated with pre-sarcopenia and sarcopenia [30]. The relatively preserved functional status of participants who were able to attend routine health examinations may also have influenced these findings, illustrating how differences in study setting and participant selection can modify observed associations.

Taken together, the clinical literature supports an association between cognitive impairment and impaired muscle health, but the extent and nature of this relationship remain heterogeneous. The association appears most evident in studies comparing individuals with more advanced cognitive impairment with cognitively normal populations. In contrast, evidence is less consistent when sarcopenia is examined as a composite diagnosis or in community-dwelling populations with relatively preserved function. Importantly, MCI represents an intermediate state of cognitive decline in which individuals have measurable impairment in one or more cognitive domains but generally retain functional independence and do not meet criteria for dementia. Accordingly, findings from studies of MCI should not be interpreted as evidence from dementia populations and are considered separately when synthesizing the clinical evidence. Longitudinal biomarker data further suggest that neurodegenerative pathology may be associated with subsequent sarcopenia progression, but these findings remain observational and do not establish a direct relationship. Moreover, the influence of age, nutritional status, physical activity, frailty, depression, and comorbidity makes it difficult to determine whether muscle decline is a direct consequence of neurodegeneration, a secondary consequence of reduced mobility and functional independence, or a manifestation of shared biological processes. Furthermore, reduced physical activity and skeletal muscle loss are not unique to dementia but are common features of normal aging and may independently contribute to sarcopenia. Other factors, including nutritional status, frailty, body composition, and comorbid conditions, may also confound or mediate the observed relationship. Overall, the available evidence generally supports an association between dementia and sarcopenia, but the predominance of cross-sectional and observational study designs limits causal inference. Consequently, the current evidence cannot determine whether dementia directly contributes to skeletal muscle decline, whether sarcopenia contributes to cognitive deterioration, or whether both conditions arise in part from shared age-related biological processes.

**Table 1:** List of the clinical studies showing the direct relation between dementia and muscle mass and function.

Study	Country	Study Design	Population (N)	Comparator	Study Focus	Sarcopenia Assessment	Key Findings	Ref
Sarabia et al., 2014	Spain	Multicenter	Dementia (189)	None	Sarcopenia prevalence and association with nutritional status	EWGSOP	68.8% had sarcopenia; malnutrition independently increased risk.	[27]
Özsürekci et al., 2019	Turkey	Cross-sectional	AD (148)	Internal comparison across AD stages	Association between sarcopenia and dysphagia across AD severity stages	EWGSOP2	Sarcopenia was associated with dysphagia; sarcopenia rates were similar across AD stages (dysphagia occurred at all stages).	[23]
Bramato et al., 2022	Italy	Cross-sectional	AD (130)	None	Diagnostic performance of sarcopenia screening tools (SARC-F-3 and MSRA-5)	EWGSOP1/2	Sarcopenia prevalence was 23.8%; both questionnaires showed poor sensitivity and were unsuitable for screening in AD.	[22]
Sugimoto et al., 2016	Japan	Longitudinal	Normal cognition, MCI, and AD (418)	Normal cognition (NC)	Relationship between cognitive decline and sarcopenia progression	EWGSOP	Sarcopenia prevalence increased progressively from normal cognition to MCI and AD (8.6% to 12.5% to 23.3%, respectively)	[25]
Kimura et al., 2018	Japan	Observational	MCI and AD (205)	Internal comparison (MCI vs. AD)	Association between nutritional factors, appetite, and sarcopenia	EWGSOP	Poor appetite and nutritional decline were associated with sarcopenia; appetite may be a modifiable risk factor.	[29]
Ceolin et al., 2025	Sweden	Population-based	Community-dwelling older adults (2291)	Community cohort	Association between AD biomarkers and sarcopenia progression	EWGSOP2	Elevated p-tau181 and NfL levels were associated with incident sarcopenia and worse sarcopenia progression over 12 years.	[26]
Ogawa et al., 2018	Japan	Cross-sectional	Normal cognition and AD (352)	Normal cognition (NC)	Sarcopenia prevalence according to AD severity	AWGS	Sarcopenia prevalence increased with AD severity (e.g., women: 11% in NC to 60% in moderate AD).	[24]
Huang et al., 2016	Taiwan	Longitudinal	Older adults (731)	Cognitively intact older adults	Association between cognitive impairment and physical function decline	Grip strength and gait speed	Sarcopenia was not associated with global cognitive impairment; low muscle strength/performance (dynapenia) were, and sarcopenia was associated with poorer verbal fluency.	[28]
Endo et al., 2021	Japan	Observational	Older adults (753)	Internal comparison (robust vs. pre-sarcopenia/sarcopenia phases)	Relationship between dementia status and sarcopenia prevalence	EWGSOP	No significant association between dementia and sarcopenia; depression was associated with pre-sarcopenia/sarcopenia.	[30]

### 3.2. The Brain-Muscle Axis: Evidence for a Shared Pathophysiology

**Figure 2:**
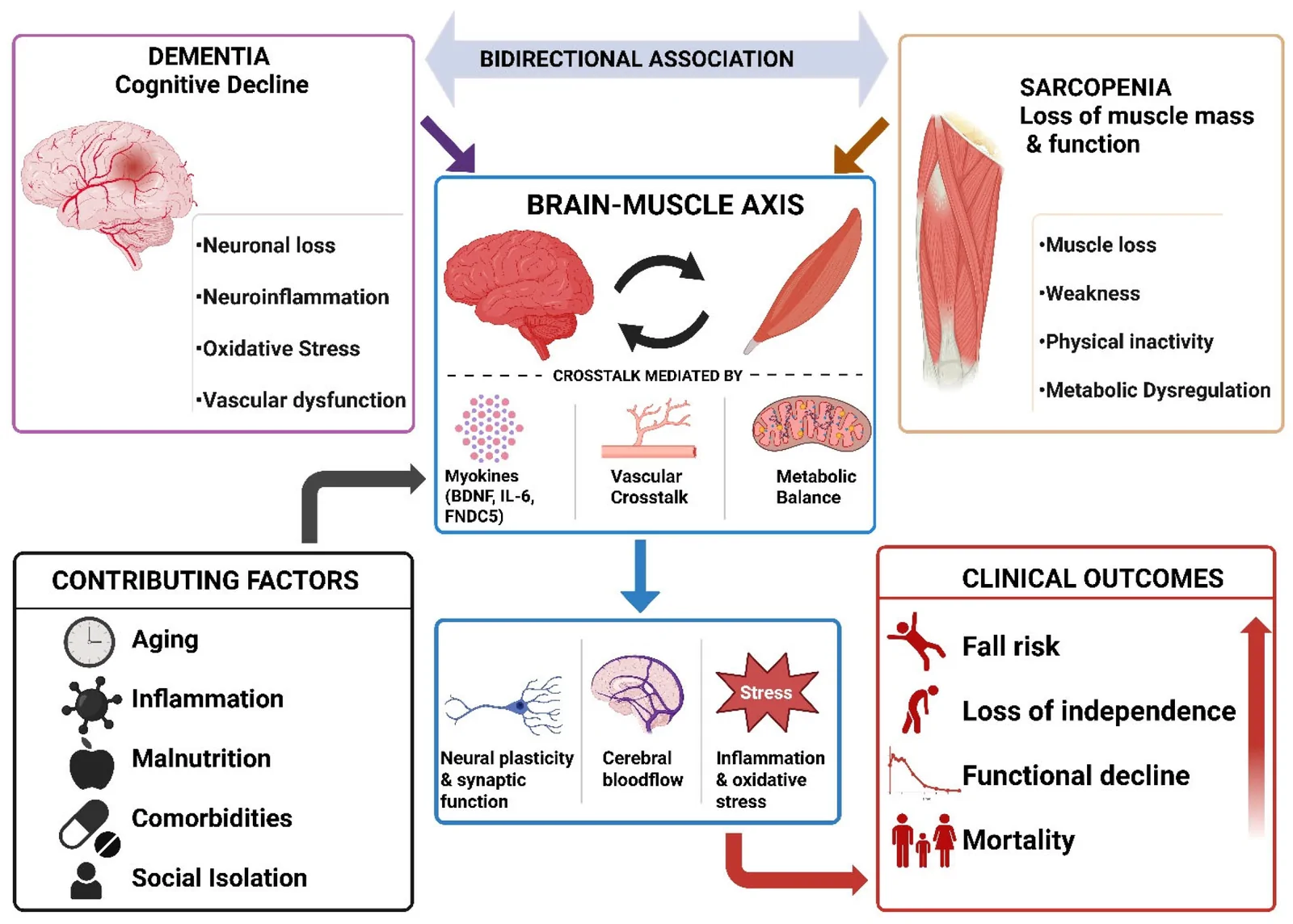
Schematic representation of the bidirectional relationship between dementia and sarcopenia through the brain–muscle axis. Neuroinflammation, oxidative stress, vascular dysfunction, and metabolic dysregulation contribute to impaired brain and muscle function, leading to functional decline, increased fall risk, loss of independence, and mortality. Contributing factors such as aging, inflammation, malnutrition, comorbidities, and social isolation further exacerbate this cycle.

The epidemiologic, clinical, and biomarker evidence supports the concept of a bidirectional brain-muscle axis (Figure 2). Dementia and sarcopenia appear to share interconnected inflammatory, metabolic, vascular, and endocrine pathways and are also influenced by aging, malnutrition, and comorbid disease. Aging and neurodegeneration are characterized by low-grade chronic inflammation. It has been reported that elevated levels of IL-6, TNF-α, and IL-1β contribute to neuronal degeneration while simultaneously impairing muscle protein synthesis and promoting muscle atrophy [31]. Mitochondrial dysfunction, oxidative stress, and impaired energy metabolism further compromise both neuronal and skeletal muscle function, providing a shared biological basis for cognitive decline and sarcopenia [32,33]. Communication between skeletal muscle and the brain is mediated in part by myokines, including irisin (FNDC5), cathepsin B, IL-6, and IGF-1, which also stimulate brain-derived neurotrophic factor (BDNF) expression. BDNF is known to promote neurogenesis and synaptic plasticity and support neuronal survival [34]. In parallel, vascular crosstalk and metabolic homeostasis influence cerebral perfusion, energy availability, and muscle function, providing additional mechanisms through which dysfunction in one organ system may adversely affect the other. Progressive muscle loss and physical inactivity may therefore diminish neuroprotective signaling, exacerbate inflammation and oxidative stress, and further accelerate cognitive decline, establishing a biologically plausible positive feedback loop.

As illustrated in Figure 2, these interacting pathways may ultimately converge on common clinical outcomes, including increased fall risk, loss of independence, functional decline, and mortality. However, most mechanistic evidence supporting this brain-muscle axis derives from experimental animal models, underscoring the need for validation in human studies. Future prospective cohort studies, randomized intervention trials, and mechanistic investigations will be essential to determine whether targeting skeletal muscle health can modify cognitive decline or whether preventing neurodegeneration can attenuate sarcopenia progression. Recognizing this relationship is important for clinical practice, as it highlights the need for integrated approaches that address both neurological and musculoskeletal health in individuals with dementia.

### 3.3. Exercise-Mediated Neurotrophic Signaling and Musculoskeletal Remodeling

**Figure 3:**
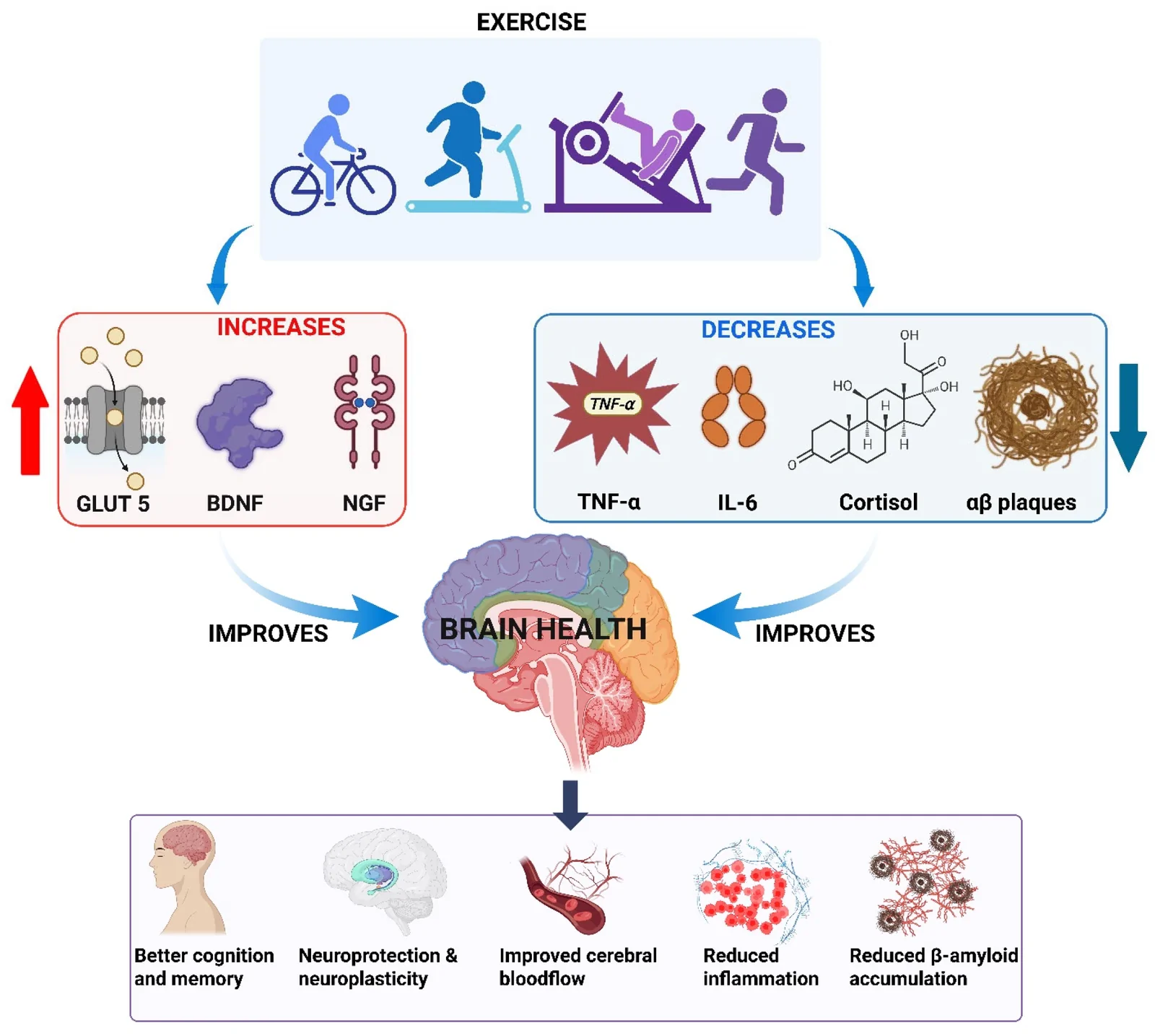
Schematic illustration of the beneficial effects of exercise on brain health. Exercise enhances neuroprotective and metabolic factors, including GLUT5, BDNF, and NGF, while reducing inflammatory mediators, cortisol levels, and β-amyloid plaque accumulation.

Building upon the mechanistic framework described above, exercise is one of the most promising non-pharmacological interventions for targeting the brain-muscle axis. Exercise can improve both muscle function and brain health through common signaling pathways. Exercise is known to reduce systemic inflammation, enhance mitochondrial function, and promote neurotrophic signaling pathways. Moreover, regular physical activity improves insulin sensitivity, vascular function, and metabolic flexibility, all of which contribute to healthier aging and may reduce vulnerability to neurodegeneration [35,36]. Exercise also modulates muscle-brain communication through the release of myokines such as cathepsin B, IL-6, and FNDC5, which promote neuronal homeostasis and synaptic plasticity [37]. Increased muscle activity stimulates the production of neurotrophic factors including BDNF and nerve growth factor (NGF), while muscle-derived lactate further enhances hippocampal BDNF expression (Figure 3) [38,39]. In parallel, exercise has been associated with improved angiogenesis and cerebrovascular health, as well as reductions in chronic inflammation, oxidative stress, cortisol levels, and markers of β-amyloid pathology [40,41]. These adaptations may support neuronal survival, preserve synaptic connectivity, and contribute to improvements in cognitive domains such as memory and executive function (Figure 3).

Importantly, exercise may also interrupt the cycle linking dementia and sarcopenia. Improvements in muscle strength and physical performance increase mobility and independence, encouraging greater physical activity and social engagement, which themselves are associated with slower cognitive decline [42]. In contrast, progressive muscle loss can contribute to increasing frailty, reduced physical activity, and worsening cognitive function. By targeting these interconnected processes, exercise may help mitigate functional decline and potentially slow the progression of cognitive and musculoskeletal impairment. However, the extent to which these effects are directly mediated through the brain-muscle axis remains uncertain.

### 3.4. Mechanistic Insights from Preclinical Models

Preclinical experimental models are well known for understanding the molecular pathways through which exercise influences neurodegeneration and skeletal muscle function. Preclinical studies suggest that exercise may contribute to the maintenance of the blood–brain barrier (BBB) integrity. In a mouse study conducted by Buttler et al. (2017), naturally hyperactive rats were shown to have increased BBB permeability and elevated angiotensin II (ANG II) levels in the brain [43]. Rats with similar exercise capacity were placed within a sedentary and training group. Those in the training group ran on a treadmill at half their exercise potential for 2 months with activity of 1 h/day for 5 days/week. Leakage of a dye known as fluorescein isothiocyanate dextran (FITC) was used as an indicator of BBB permeability, and a large reduction in leakage was observed after the training experiment. Similarly, He et al. (2017) investigated the impact of running exercise on cognition and spatial memory, focusing on its effects on the glymphatic pathway and solute fluid clearance [44]. Mice were divided into two groups: one without exercise and another with voluntary access to a running wheel (16 cm diameter) for 6 weeks. FITC dye was injected into the cisterna magna, and the bloodstream was labeled with RD. The study reported significantly lower tracer levels at 30 min (*p* < 0.05), 45 min (*p* < 0.01), and 60 min (*p* < 0.001) in the exercise group. This indicated reduced dye extravasation and was interpreted as evidence of improved glymphatic clearance in the exercised mice. Additionally, clearance of amyloid plaque deposition was assessed by measuring the pixel intensity of Aβ1-16, which was markedly lower in the running group (20.67) compared to the control group (61.5, *p* < 0.001).

Neeper et al. (1996) [45] performed a study on adult male rats. They compared levels of BDNF and nerve growth factor (NGF) in adult male rats after 0, 2, 4, and 7 nights of access to a running wheel [45]. The study found that by night 7, BDNF mRNA levels had notably increased in the hippocampus and the posterior third of the cerebral cortex compared to levels observed on night 2, suggesting that exercise can increase BDNF transcription in this experimental model. Similarly, a study by Baranowski et al. (2021) investigated the impact of BDNF levels on beta-site amyloid precursor protein cleaving enzyme 1 (BACE1) and its role in the production of beta-amyloid plaques [46]. Twelve inactive mice were placed on a treadmill and ran at a 15 m/min speed for 120 min. BDNF levels in the hippocampus and prefrontal cortex were measured two hours after the exercise. They found a significant increase in BDNF levels (*p* < 0.05) and a reduction of BACE1 in the prefrontal cortex. Given that BACE1 is essential for amyloid-β production, its downregulation suggests a potential reduction in β-amyloid formation [46]. Zhang et al. (2022) examined 10-month-old APP/PS1 transgenic mice and wild-type controls, which were divided into inactive groups and those given access to a running wheel for 3 months [47]. Using ELISA, the study assessed levels of GLUT5, a regulator of microglial activity, and TREM2 (Triggering Receptor Expressed on Myeloid cells 2), a microglial surface receptor involved in neuroinflammation and neurogenesis. Exercise was associated with significant increases in TREM2 (*p* = 0.012) and GLUT5 (*p* = 0.017), suggesting potential effects on microglial function and neuroimmune signaling.

Collectively, these preclinical experimental studies provide mechanistic evidence that exercise can modulate multiple biological pathways involved in neurodegeneration, including blood–brain barrier integrity, glymphatic clearance, neurotrophic signaling, and neuroimmune regulation. While these findings support biological plausibility for exercise-related neuroprotective effects, translation of these mechanisms into clinical benefit requires confirmation in human studies.

### 3.5. Translational Evidence for Exercise-Induced Neuroprotection

Consistent with pre-clinical experimental models, human studies also demonstrate that regular physical activity is associated with improvements in cognitive performance. Although clinical studies cannot always establish the underlying molecular mechanisms, they provide important evidence that exercise-related adaptations may translate into measurable functional benefits. A recent study reported that individuals involved in regular aerobic exercise, such as 40 min of walking at 50–75% of maximal heart rate, demonstrated increased hippocampal blood flow and volume, along with improved memory performance and cognitive test scores. These changes were also accompanied by elevated levels of BDNF, further supporting exercise-induced neuroprotection [48].

A randomized clinical trial conducted in Shanghai (China) by Mortimer et al. (2012) involved 120 older adults who were randomly assigned to one of four groups: Tai Chi, walking, social interaction, or no intervention, over 40 weeks [49]. Brain volume was assessed using MRI before and after the intervention. Participants in the Tai Chi and social interaction groups showed significant increases in overall brain volume compared with the control group (*p* < 0.05). In a separate study, Law et al. (2018) examined 85 cognitively normal adults with a mean age of 64 years [50]. Physical activity levels were monitored using accelerometers to capture both intensity and duration of movement. Followed by the collection of cerebrospinal fluid to analyze Aβ42, total tau, and phosphorylated tau. They observed that lower Aβ42 levels and higher tau/Aβ42 ratios are associated with greater disease burden. The results showed that individuals with moderate-to-high physical activity had higher Aβ42 levels and lower tau/Aβ42 ratios, suggesting a more favorable biomarker profile. Those with little spontaneous movement had a lower rate of Aβ42 (*p* = 0.014). These findings indicate an association between higher physical activity and more favorable cerebrospinal fluid biomarker profiles, but do not establish that exercise directly increases amyloid clearance or prevents amyloid accumulation [50].

Alghadir et al. (2021) studied the effect of exercise on IL-6, cortisol, TNF-α, CRP, and cognitive health after 12 weeks of exercise [51]. The study reported significant reductions in inflammatory biomarkers, including IL-6, TNF-α, and C-reactive protein (CRP), alongside improvements in cognitive test scores in individuals with MCI (*p* < 0.01). Collectively, these clinical studies demonstrate that exercise is associated with improved cognitive performance and favorable changes in inflammatory and neurodegenerative biomarkers across diverse exercise modalities. Despite differences in exercise type, duration, and intensity, these findings support the potential translational relevance of exercise-induced biological adaptations observed in experimental models, while recognizing that clinical studies do not necessarily establish the underlying mechanisms or causality.

Overall, evidence from human and animal studies supports exercise as a potential modulator of the brain-muscle axis through coordinated molecular and physiological adaptations. Experimental models demonstrate that exercise promotes neuroprotection through increased BDNF signaling, improved glymphatic clearance, reduced β-amyloid accumulation, and modulation of systemic inflammatory pathways. Clinical studies complement these findings by demonstrating improvements in cognitive performance, brain structure, and inflammatory and neurodegenerative biomarkers following exercise interventions. Exercise-induced myokines, together with improvements in mitochondrial function and metabolic homeostasis, provide a biologically plausible link between skeletal muscle activity and brain health, supporting the role of physical activity in maintaining cognitive resilience and potentially slowing age-related neurodegenerative decline. Limitations included generalizability primarily for individuals aged 60 years and older with dementia. Only studies published in English were included, introducing potential language bias. Additionally, heterogeneity in study design and variability in dementia severity, including populations with MCI, may have influenced the overall findings.

## 4. Conclusions

The recent literature supports a strong correlation between various types of dementia and the progression of sarcopenia. The studies also reported a higher prevalence of sarcopenia in males compared with females, suggesting possible sex-related differences in vulnerability, potentially mediated by hormonal or disease-specific factors. However, current evidence is insufficient to define the biological basis of these differences, and further sex-stratified studies are required. Nutritional status and frailty also appear to play important contributory roles, with poorer dietary intake associated with higher sarcopenia prevalence in individuals with cognitive impairment. At the same time, comorbid conditions such as hypertension, cerebrovascular disease, and depression likely contribute to an interconnected network influencing memory loss and muscle deterioration. However, depression alone does not appear to alter the observed association significantly. Future studies should integrate clinical and experimental approaches, including longitudinal human cohorts and animal models, with skeletal muscle transcriptomic and molecular profiling to better elucidate pathways involved in dementia and sarcopenia. Such studies are essential to clarify whether observed muscle decline reflects shared upstream mechanisms or secondary effects of neurodegeneration. Importantly, the evidence also supports exercise as a key modifiable intervention, with consistent benefits on cognitive function and muscle health via improved neuroplasticity, increased BDNF expression, reduced neuroinflammation, improved glymphatic clearance, and decreased β-amyloid accumulation. Furthermore, exercise-induced myokine signaling reinforces the biological coupling between skeletal muscle activity and brain health, supporting a potential role for exercise in attenuating neurodegenerative progression in Alzheimer’s disease.

## Data Availability

No new data were created or analyzed in this study. Data sharing is not applicable to this article.

## Abbreviations

AD	Alzheimer’s disease
AWGS	Asian Working Group for Sarcopenia
EWGSOP	European Working Group on Sarcopenia in Older People
MCI	mild cognitive impairment
NfL	neurofilament light chain
NC	normal cognition
p-tau181	phosphorylated tau-181
